# Comprehensive analysis of the functional microRNA–mRNA regulatory network identifies miRNA signatures associated with glioma malignant progression

**DOI:** 10.1093/nar/gkt1054

**Published:** 2013-11-03

**Authors:** Yongsheng Li, Juan Xu, Hong Chen, Jing Bai, Shengli Li, Zheng Zhao, Tingting Shao, Tao Jiang, Huan Ren, Chunsheng Kang, Xia Li

**Affiliations:** ^1^College of Bioinformatics Science and Technology, Harbin Medical University, Harbin 150080, China, ^2^Department of Neurosurgery, Tiantan Hospital, Capital Medical University, Beijing 100050, China, ^3^College of Fundamental Medical Sciences, Harbin Medical University, Harbin 150080, China and ^4^Department of Neurosurgery, Tianjin Medical University General Hospital, Laboratory of Neuro-Oncology, Tianjin Neurological Institute, Laboratory of Neurotrauma, Variation and Regeneration, Ministry of Education and Tianjin Municipal Government, Tianjin 300052, China

## Abstract

Glioma is the most common and fatal primary brain tumour with poor prognosis; however, the functional roles of miRNAs in glioma malignant progression are insufficiently understood. Here, we used an integrated approach to identify miRNA functional targets during glioma malignant progression by combining the paired expression profiles of miRNAs and mRNAs across 160 Chinese glioma patients, and further constructed the functional miRNA–mRNA regulatory network. As a result, most tumour-suppressive miRNAs in glioma progression were newly discovered, whose functions were widely involved in gliomagenesis. Moreover, three miRNA signatures, with different combinations of hub miRNAs (regulations≥30) were constructed, which could independently predict the survival of patients with all gliomas, high-grade glioma and glioblastoma. Our network-based method increased the ability to identify the prognostic biomarkers, when compared with the traditional method and random conditions. Hsa-miR-524-5p and hsa-miR-628-5p, shared by these three signatures, acted as protective factors and their expression decreased gradually during glioma progression. Functional analysis of these miRNA signatures highlighted their critical roles in cell cycle and cell proliferation in glioblastoma malignant progression, especially hsa-miR-524-5p and hsa-miR-628-5p exhibited dominant regulatory activities. Therefore, network-based biomarkers are expected to be more effective and provide deep insights into the molecular mechanism of glioma malignant progression.

## INTRODUCTION

Glioma is the most common and malignant brain cancer, and ∼10 000 new cases of high-grade or malignant glioma occur each year (1). Despite advances in treatment modalities, extremely poor prognosis of glioma remains unchanged over the last three decades (2). It is an urgent clinical challenge to identify sensitive and specific early biomarkers for diagnosis and prognosis as well as for the investigation of the mechanisms underlying the development and progression of glioma.

MicroRNAs (miRNAs) are small non-coding RNAs that regulate gene expression post-transcriptionally and play important roles in regulating diverse biological processes (3). During the initiation and progression of human cancers, miRNAs have been shown to modulate cell proliferation, survival, tumour angiogenesis, invasion and metastasis (4,5), and dysregulation of miRNA also has been observed in various types of human cancers, including gliomas (6,7). However, our understanding of miRNA expression patterns as potential biomarkers for diagnosis, prognosis, disease progression and personalized therapy is just emerging. For example, Rao *et al.* (8) identified a 23-miRNA expression signature that can discriminate anaplastic astrocytoma from glioblastoma (GBM) by profiling 39 malignant astrocytoma and seven normal brain samples. In addition, Zhang *et al.* (9) and Srinivasan *et al.* (10) both found that the signature consisting of several miRNAs could predict GBM patient survival. However, few of these studies attempted to profile miRNA expression in different grades of glioma, and the mechanism in the progression of glioma largely remains unknown.

Although multiple miRNA biomarkers have been identified in glioma, especially in GBM, the functions of most miRNAs remain unknown because of lack of candidate target genes under the specific biological conditions. The biologically relevant targets of each miRNA may vary from one tissue to another, whereas many genes contain putative binding sites for multiple miRNAs. Based on the wide acceptance that miRNAs reduce, at least partially, the expression level of targeted genes (11–13), integration of predicted miRNA–target regulations with both miRNA and mRNA expression gives us a chance to identify the underlying target genes whose expressions are inversely regulated by miRNAs in a specific condition. Increasing studies highlight the success of this strategy (14,15). In addition, a single miRNA can regulate many target genes in mammalian cells and multiple miRNAs may regulate the same targets. To understand the complex regulatory relationships, attempts have been made to investigate miRNA-mediated regulation by biological network analyses (14,16,17). For example, Yang *et al.* (18) recently uncovered a miRNA-regulatory network, and found that eight key miRNAs could negatively regulate most signature genes, which could define two subtypes associated with poor overall survival of women with serous ovarian cancer. However, glioma progression-associated miRNA–mRNA regulatory network is blocked by the lack of the expression of miRNAs and mRNAs that are simultaneously profiled across different grade samples. Furthermore, our understanding of the glioma progression-associated miRNAs is just emerging.

To investigate this issue, we conducted an extensive miRNA profiling and mRNA profiling study on a cohort of 160 glioma patients with different grades in China. These data and miRNA–target regulation information were integrated, and a multi-step approach was proposed to construct the functional miRNA–mRNA regulatory network (FMRN) associated with glioma malignant progression. Here, we expected that a gene is regulated by a miRNA, only if the miRNA has predicted binding sites in this gene and that their expressions are negatively correlated. Then, we uncovered that several key miRNAs regulating most targets in the FMRN not only play important roles in glioma malignant progression, but also can independently predict the survival of patients with different glioma grades. The integrative analyses provided novel insights into regulatory mechanisms at the miRNA level during the progression of glioma, and both the method and predictions generated here could serve as important resources for future experimental dissection of miRNA functions in glioma.

## MATERIALS AND METHODS

### Materials

#### Clinical sample characteristics

A retrospective series of 160 glioma patients received from Beijing Tiantan Hospital was considered, and the extended demographics are provided in Table 1, Supplementary Table S1 and Supplementary materials. All these samples were histologically graded according to current WHO classification of tumours of the nervous systems, including 63 WHO grade II patients, 33 grade III patients and 64 GBM patients. Written informed consent was obtained from all donors. Clinical investigations were conducted after approval by the local research ethics committee and in accordance with the ethical principles.

**Table 1.** Clinicopathologic characteristics of patients with glioma in the CGGA cohort (*n* = 160)

Characteristics	Number of patients			*P*
	All patients	Training set	Test set	
	*N* = 160	*N* = 80	*N* = 80	
Stage				0.99^a^
II	63	32	31	
III	33	16	17	
IV	64	32	32	
Sex				0.99^a^
Female	64	32	32	
Male	96	48	48	
Age				0.74^b^
Mean±SD	41.19 ± 12.50	41.49 ± 13.16	40.90 ± 11.89	
Range	12–70	12–70	17–65	
*IDH1* mutation				0.73^a^
Mutated	57	27	30	
Wild	74	38	36	
Survival (month)				0.86^b^
Mean±SD	22.67 ± 11.10	22.84 ± 11.92	22.49 ± 10.27	
Range	3.47–55.27	3.47–55.27	4.83–43.47	
State				0.42^a^
Living	68	31	37	
Death	92	49	43	

*IDH1*, isocitrate dehydrogenase 1; SD, standard deviation.

^a^*P*-values were determined using Fisher’s exact test.

^b^*P*-values were determined using Student’s *t*-test.

#### Genome-wide mRNA and miRNA expression profiling

Matched genome-wide miRNA and mRNA expression profiling was successfully obtained from these 160 glioma samples by using human v2.0 miRNA expression BeadChip and Agilent Whole Human Genome Array (see Supplementary materials). All data were deposited in Chinese Glioma Genome Atlas (CGGA), a publicly available database that focuses on glioma. The expression values for each gene were background-subtracted, quantile-normalized and log2-transformed, whereas the miRNA expressions were also log-transformed. The miRNA (or gene) probes were filtered to remove non-specific probes and probes lacking association with miRBase entry IDs (or Entrez Gene IDs) (19), and, in turn, if multiple probes were corresponding to a single miRNA (or a gene), the expression values of these redundant probes were averaged. Lastly, 818 miRNAs and 18 634 genes were analysed.

#### miRNA target prediction

The regulatory relationships between miRNAs and mRNAs were considered as the combination of all predicted targets from four major algorithms: TargetScan5.1 (20), miRanda (miRBase version 5) (19), Pictar (four-way) (21), and DIANA-microT (version 3.0) (22) (further details in Supplementary materials). Here, we built on previous results and particularly on the conclusion common to multiple studies that had used the combination of the predictions from these algorithms rather than the intersection, which provides a more promising strategy to select true targets (23,24).

### Methods

#### Identification of miRNAs and genes associated with glioma malignant progression

Differential expression analysis was performed to identify miRNAs (or genes) associated with the malignant progression of glioma by respectively comparing the miRNA or gene expressions in grade III or grade IV gliomas with those in grade II gliomas. An miRNA or gene was defined to be associated with the malignant progression of glioma, only if it showed differential expression in both conditions. Differential expression was detected by unpaired Student’s *t*-test. The false discovery rate (FDR) was controlled by Benjamini and Hochberg algorithm (both *q*-values at 5% thresholds) (25).

#### Construction of the FMRN by the integrative computational method

To identify the functional regulations from miRNAs to mRNAs, we combined both the computational target predictions at the sequence level and the inverse expression relationships between miRNAs and mRNAs in the context of glioma progression (Supplementary Figure S1A). Specifically, the functional regulations were detected using the subset of miRNAs and genes characterized by (i) both miRNAs and genes associated with glioma malignant progression, (ii) a regulatory relationship according to computational predictions, and (iii) expression profiles strongly anti-correlated. Indeed, as miRNAs tend to down-regulate target mRNAs, the expression profiles of genuinely interacting pairs were expected to be anti-correlated. Thus, the Pearson correlation coefficients were calculated for each predicted miRNA–mRNA pair, whose threshold was set to <−0.4 and corresponding *P* value was set to <0.05. Finally, after assembling all functional miRNA–target gene pairs, we generated the FMRN, which is associated with glioma malignant progression. This network is a bipartite graph containing two sets of vertices (nodes) corresponding to miRNAs and target genes. A directed edge (connection) from a miRNA to one of its targets exists if their relationship is functionally regulated. To facilitate analysing the topological structure of the FMRN and identifying miRNAs playing central roles in the FMRN by the well-used measure—degree, the weights of all edges were set to 1.

#### Unsupervised hierarchical clustering

To visualize the expression pattern of miRNAs and target genes in the FMRN, unsupervised hierarchical clustering analysis was carried out, respectively. Prior to the hierarchical clustering, all samples were ranked according to their grades. Hierarchical clustering of the expression profile was done using average linkage and 1-Pearson correlation as a distance measure.

#### Development and validation of the miRNA risk scoring system

To construct and validate three miRNA signatures that can respectively predict survival of patients suffering from glioma, high-grade glioma as well as GBM, the considered specimens were randomly assigned to a training data set or a test data set. Two sample subsets were required to have the same size. In the random assignment of glioma or high-grade glioma patients, the grade information was also considered to make sure that there was no grade difference between the training and test subsets. According to previous studies (26,27), we used the splitting strategy as opposed to cross-validation, because there was no overlap between the two sample sets, which was an advantage over cross-validation.

We aimed to identify three clinically significant prognostic miRNA signatures from three training sets and tested them using the corresponding internal validation sets. The miRNAs playing central roles in the FMRN, miRNA hubs, were considered as the candidate members of the signatures. The hubs were commonly defined as the top 15% of the nodes by degree (28–30), in this case, corresponding to miRNAs regulating at least 30 genes in the FMRN. Totally, there were 21 hub miRNAs for further analysis. Then, we used univariate Cox regression analysis to evaluate the association between survival and the expression level of each hub miRNA. Regression coefficient with a plus sign indicated that increased expression is associated with an increased risk of survival (risky miRNAs), and, conversely, a minus sign indicated that increased expression is associated with a decreased risk of survival (protective miRNAs). After selecting hub miRNAs that were significantly correlated with survival (*P* < 0.05), a mathematical formula for survival prediction was constructed, taking into account both the strength and positive or negative association of each miRNA with survival. More specifically, we assigned a risk score to each patient according to a linear combination of the miRNA-expression values weighted by the regression coefficients from the aforementioned univariate Cox regression analysis. The risk score for each patient was calculated as follows:

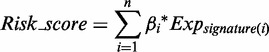

where 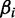 is the Cox regression coefficient of hub miRNA *i* in the training set, and *n* is the number of miRNAs that are significantly associated with survival. All patients in the training data set were thus dichotomized into high-risk and low-risk groups using the median risk score as the cut-off point. Patients having higher risk scores were expected to have poor survival outcomes. The coefficient and threshold values derived from three training sets were directly applied to miRNA expression data of the corresponding test sets to divide the patients in the test sets into high-risk and low-risk groups. The Kaplan–Meier method was further used to estimate the overall survival time for the two subgroups. The differences in the survival times were analysed using the log rank test. The workflow was shown in Supplementary Figure S1B.

#### Randomization tests

To evaluate whether our integrated approach in identifying the survival-associated miRNA signatures is better than the traditional approach only based on miRNA expression, we firstly ranked miRNAs based on the FDR in an ascending order, and chose the top 21 miRNAs with the most differential expression as candidate miRNAs to evaluate their prognostic performance in the training and test samples. Furthermore, to determine the significance of each miRNA signature, we performed three different randomization tests by randomly choosing miRNAs from three different backgrounds: non-hub miRNAs in the FMRN, miRNAs differentially expressed in grade III and IV, when compared with grade II glioma, and miRNAs assayed in microarray. The first type of randomization considered miRNA topological feature in the FMRN. For each kind of randomization test, we randomly selected 21 miRNA candidates from the background at a time, the miRNA signature was detected and its effectiveness for clinical outcome prediction in the training and test data set were computed based on the aforementioned risk scoring system. The corresponding *P* values of log-rank tests were respectively calculated in the training and test data sets, and the negative log-transformed *P* values were used as evaluation indexes. The higher these two scores, the better was the performance of a miRNA signature. We repeated each procedure 1000 times, and the significance was defined as the proportion of times in which in random conditions, the *P* values for training and test sets were both lower than the real ones.

#### Functional analysis of miRNAs via their targets in the FMRN

To explore the functional roles of miRNAs in two disparate subnetworks of the FMRN as well as the identified miRNA prognostic signatures in high-grade glioma and GBM, we respectively used their regulatory gene subsets to perform functional enrichment. This process was realized by the tool WebGestalt (31), using the human genome as the reference set and the hypergeometric test to calculate the enrichment significance. KEGG pathways or Gene Ontology terms were considered. If the whole genome had a total of *N* genes, of which *K* were involved in the function category under investigation, and the set of interesting target genes for analysis had a total of *M* genes, of which *x* were involved in the same function category, then the *P* value can be calculated to evaluate the enrichment significance for that function category as follows:

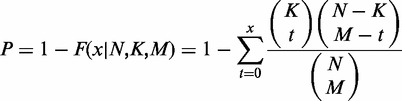



Then, the enriched significance *P* values were adjusted by Benjamini and Hochberg method and finally, Kyoto Encyclopedia of Genes and Genomes (KEGG) pathways (or GO terms) with adjusted *P* values < 0.01 and including at least two interesting genes were considered.

## RESULTS

### Global properties of FMRN constructed by integrative genomics

**Figure 1.**
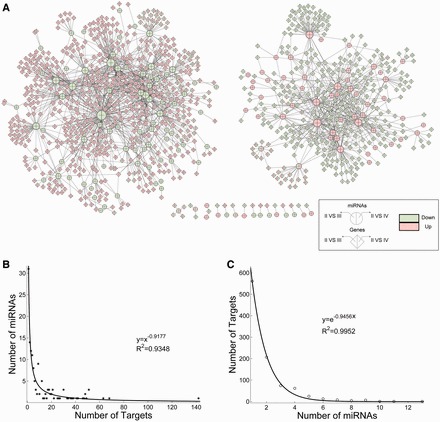
The layout of the FMRN and its structural features. (**A**) The FMRN generated by the procedure described in the Materials and Methods. This network consists of 1841 regulations between 145 miRNAs and 965 genes. A circle marks miRNA and a diamond marks the gene. An edge represents a regulation from miRNA to one of its targets in the context of glioma progression. The miRNAs and genes are colored based on their dysregulation direction. If miRNAs (or genes) are highly expressed in grade III glioma than in grade II glioma, the left part of nodes are marked in red, otherwise they are marked in green. Likewise, if miRNAs (or genes) are highly expressed in grade IV glioma, they are also marked in red in the right parts; otherwise, they are marked in green. (**B**) Out-degree distribution of the FMRN. Most of the miRNAs are lowly connected and only a few are relatively highly connected. The examination of the out-degree distribution of the FMRN revealed a power-law with a slope of −0.9177 and *R^2^* = ∼0.9348. (**C**) In-degree distribution of the FMRN. In-degree is defined as the number of regulatory miRNAs for each target, signifying an exponential distribution with an exponent of −0.9456 and *R^2^* = ∼0.9952.

To identify miRNAs and genes that are likely to play important roles during glioma malignant progression, differential expression analysis was performed by respectively comparing their expressions in grade III or IV gliomas with those in grade II gliomas. Firstly, 344 miRNAs and 6338 coding genes, which showed significantly differential expressions when comparing grade III or IV glioma samples with grade II glioma samples were obtained in the microarray analysis, suggesting the widespread changes in both miRNA and mRNA expressions in the glioma malignant progression. Then, we adopted an integrated approach to construct the FMRN by combining two independent, yet complementary, types of information: inverse expression relationships and computational target predictions. It has been suggested that the regulatory relationships identified using these two sources of information are more likely to be physiologically functional than those identified using either one of them alone (17,32). By further investigating their target relationships and negative correlations of expression, all significant miRNA–mRNA pairs were identified and assembled into the FMRN, which consisted of 1841 regulations between 145 miRNAs and 965 genes (Figure 1A, Supplementary Table S2). Most of these regulations connected together and formed two big subnetworks (Figure 1A). By further investigating the expression change in miRNAs and genes in the network, we found that the expression of all miRNAs in the left subnetwork consistently decreased in grade III and IV glioma when compared with grade II glioma; meanwhile, in the right subnetwork, 50 of 52 miRNAs exhibited consistent increased expression. On the other hand, 88.6 and 90.6% target genes in the left and right subnetwork also respectively exhibited consistent expression deregulation. Thus, the consistent expression deregulation and negative functional regulations might be the main reasons for the formation of two disparate subnetworks. These results indicated that these subnetworks indirectly reflect the expression patterns of miRNAs and genes, and that understanding the structure of the FMRN could provide more insights into the tumourigenesis of glioma.

Moreover, we found that 79.2% of the differentially expressed miRNAs regulate at least two targets, and ∼41.8% mRNAs are co-regulated by two or more miRNAs. These results indicated a complicated combination in terms of both target multiplicity and miRNA cooperatives in the progression of glioma. Then, we evaluated the ‘out-degree’ of miRNAs, defined as the number of outgoing edges per node. We found that most miRNAs are lowly connected and only a few are relatively highly connected, as shown in Figure 1A and B. The examination of the out-degree distribution of the FMRN revealed a power-law with a slope of −0.9177 and *R^2^* = 0.9348. We also calculated the ‘in-degree’ of each target gene, which is the number of incoming edges. The ‘in-degree’ distribution signifies an exponential distribution with an exponent of −0.9456 and *R^2^* = 0.9952 (Figure 1C). These results suggested that similar to many biological networks, the FMRN is not random but characterized by a core set of organizing principles in structure that distinguishes it from random networks. Understanding the progression of glioma in the context of these network principles could allow us to address some fundamental properties of glioma miRNAs.

### Differentially expressed miRNAs in glioma progression control broad biological functions

**Figure 2.**
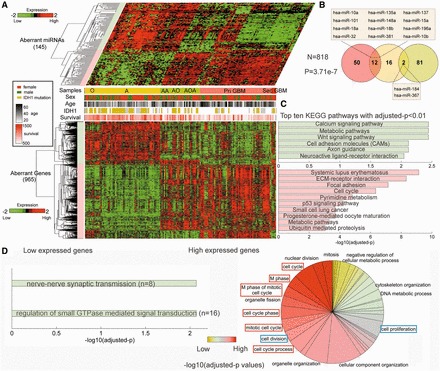
The expression patterns and functions of miRNAs in the FMRN. (**A**) Expression patterns of miRNAs and their regulatory targets in the FMRN using one-dimensional hierarchical clustering analysis. The miRNAs and genes are globally grouped into two classes, and the corresponding distance thresholds for clustering analysis were 0.90 and 0.85 for miRNAs and genes, respectively. Low-expressed miRNAs in high-grade glioma are located in the left subnetwork of the FMRN, while high-expressed ones are in the right subnetwork. The genes exhibit the reverse trend. (**B**) The intersection between known glioma miRNAs and two miRNA sets in separate subnetworks of the FMRN. The miRNAs in the left subnetwork are rarely found to be involved in gliomagenesis. The red circle represents the up-regulated miRNAs and green circle represents the down-regulated miRNAs, and inter yellow circle represents the known glioma miRNAs. (**C**) and (**D**) Functional enrichment analysis of two sets of target genes, indicating the functional roles of two miRNA sets in different subnetwork of the FMRN. (**C**) Enrichment results for pathways analysis; the pathway information was obtained from the KEGG database. Red color corresponds to up-regulated genes and green color corresponds to down-regulated genes. (D) Enrichment results for biological processes; this information was obtained from the GO database. The orders of biological processes listed in the circle are based on their enriched significance. Red color marks the most enriched functions, whereas green color corresponds to the lowest enriched ones. The area is in accordance with the number of targets annotated in the biological process.

Next, we investigated the expression patterns of miRNAs and their target genes in the FMRN, and hierarchical clustering analysis revealed that both miRNAs and genes were globally grouped into two classes. In each class, their expressions were similar across all patient samples, even in different grade glioma (Figure 2A). We further found that the classifications of miRNAs and genes based on clustering are in accordance with their locations in the FMRN. As shown in Figure 2A, miRNAs in the left subnetwork were all included in the upper expression cluster of miRNAs, which represented down-regulated miRNAs in glioma progression with suppressive progression potential. Correspondingly, their regulatory target genes were mainly grouped into the highly expressed gene clusters in high-grade gliomas. The miRNAs and their target genes in the right subnetwork of the FMRN showed the inverse trend. Increasing evidences have revealed that miRNAs play important roles in many key biological processes, and some researchers have manually retrieved the associations of miRNAs and diseases from literature data. Thus, we obtained experimentally confirmed glioma-related miRNAs from two common databases: miR2Disease (33) and HMDD (34). The known glioma-related miRNAs are commonly identified by comparing with normal tissues and mainly associated with GBM, and, in this study, we further explored their expression patterns in glioma progression. After comparing these known glioma miRNAs with miRNAs in the FMRN, we found that many known glioma miRNAs are included in the FMRN, and especially most are located in the right subnetwork (Figure 2B, *P* = 3.71e-7, hypergeometric test), showing over-expression during glioma progression. In contrast, the left subnetwork of the FMRN includes relatively more miRNAs that were not found to be involved in glioma, which might be considered as novel candidate disease ones. Their dysregulation directions during progression imply their suppressive progression potential. However, their functions in glioma are still largely unknown, let alone glioma progression. Fortunately, enrichment analysis of the target genes of these miRNAs in the FMRN could give us a global clue of their functional roles in the progression of glioma. Functional enrichment analysis was respectively carried out for the target genes of miRNAs in each subnetwork of the FMRN. As a result, miRNAs in two subnetworks significantly participated in several biological pathways directly relevant to gliomas and neurodevelopment (Figure 2C), indicating that they might have important biological implications in glioma oncogenesis. For example, low-expressed miRNAs in high-grade glioma, which included relatively more novel candidate disease miRNAs, controlled many functions directly associated with cancers (p53 signaling pathway, cell cycle and Focal adhesion). These results suggested the potential regulatory roles of these low-expressed miRNAs with no previously reported involvement in glioma. On the other hand, among the pathways enriched by genes in the right subnetwork of the FMRN, we concentrated on calcium signalling pathway, metabolic and Wnt signalling pathways (adjusted *P*-values < 3.60e-3). Calcium signal is the most common way of transmitting signals from cell environment to the cytoplasmic calcium binding effectors, and has broad effect on cell behavior. Being a crucial player in neuronal transmission, increasing studies have shown that it is important for glia physiology (35).

In addition, we also identified GO terms that were significantly enriched by target genes of miRNAs in the two subnetworks. Overall, we found that only two GO terms are significantly regulated by miRNAs in the right subnetwork, which are also associated with neurodevelopment. On the other hand, the low-expressed miRNAs specially participated in the cell cycle, cell division and cell proliferation-related processes (Figure 2D). Multiple steps of cell cycle were found to be involved, and evidences had shown that dysregulations of the cell cycle components may lead to tumour formation (36,37).

These results suggest that differentially expressed miRNAs in glioma progression control broad biological functions associated with cancers. Moreover, we identified many novel disease miRNAs with suppressive progression potential, which significantly control many important biological functions, especially multiple steps of cell cycle. Therefore, integrative analyses of the miRNA and gene expression data can reveal the dysregulated functions underlying the progression of glioma.

### Hub miRNAs in the FMRN stratify glioma/high-grade glioma patients into different risk groups

**Figure 3.**
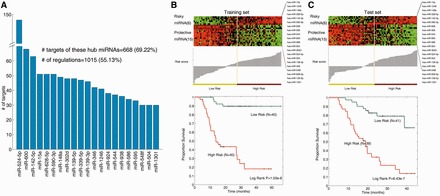
The expression pattern of hub miRNAs and their performance in survival analysis based on all glioma patients. (**A**) A histogram revealing the number of targets for each of the 21 hub miRNAs in the FMRN. (**B**) (Upper panel) Colour-gram of miRNA expression profiles of glioma patients in the training set and the miRNA risk-score distribution. Rows represent high-risk and protective miRNAs and columns represent patients. The yellow line represents the median miRNA signature cut-off dividing patients into low-risk and high-risk groups. (Bottom panel) Kaplan–Meier estimates of overall survival of glioma patients according to the miRNA signature. (**C**) Eighty glioma patients in the test data set.

It is known that hubs (nodes mainly making links in a network) are a central part of network, important to the stability of the biological system. Likewise, miRNAs regulating more targets in the FMRN might play more important roles in glioma malignant progression and influence more critical functions. Thus, it is interesting to study the efficiency of hub miRNAs as prognostic signatures. Hubs are commonly defined as the top 15% of the nodes by degree (11,26,28), corresponding to miRNAs regulating ≥30 target genes in our study. Thus, we specifically abstracted the top 21 hub miRNAs. These 21 hub miRNAs regulated ∼70% of the targets in the FMRN, and were involved in more than half of the regulations of the total network (Figure 3A), implying central roles of these miRNAs in glioma. Subsequently, we examined whether these hub miRNAs are associated with the survival of glioma patients. After randomly assigning the 160 specimens to a training set (*n* = 80) or a test set (*n* = 80), we found that the clinical characteristics had no significant difference between these two sets (Table 1). Next, we only used the training data set for detecting the miRNA signature of glioma. As a result, these hub miRNAs were all found to be significantly associated with glioma patient survival, constructing a risk signature. Among them, 15 miRNAs were protective, and the other six miRNAs were risky (Supplementary Table S3). By using the miRNA risk scoring system as described in the method section, patients in the training set were further divided into high- and low-risk groups based on their risk scores. Consequently, patients with high-risk scores had shorter median survival than those with low-risk scores (Figure 3B, *P* = 1.03e-8); the median survival time of high-risk group was 13.78 months and that of low-risk group was 29.25 months. Next, we validated the miRNA signature in the test set where samples were also classified into the high-risk group or low-risk group using the same cut-off point as in the training set. Similarly, patients with high-risk score also had shorter median overall survival than patients with low-risk score (Figure 3C, *P* = 6.43e-7). To further ascertain whether the risk signature comprising of hub miRNAs is an independent predictor of glioma patients’ survival, the prognostic association between our newly identified hub miRNA signature and other known clinical and pathologic risk factors for glioma progression was assessed by univariate and multivariate analyses. As expected, in addition to patient age and *IDH1* mutation status, which are already well-known risk factors, the 21-hub miRNA signature was a significant risk factor for survival in univariate analysis (Supplementary Table S4). Multivariate analysis further revealed that the miRNA signature remained an independent prognostic risk factor for glioma patient survival (*P* = 1.12e-6; see details in Supplementary Table S4), although isocitrate dehydrogenase (IDH) mutation status had weak significance (*P* = 0.063).

**Figure 4.**
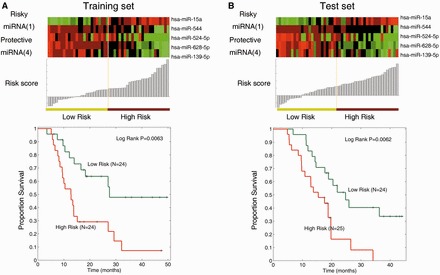
A five-miRNA signature of high-grade gliomas. (**A**) Forty-eight high-grade glioma patients in the training data set. (**B**) Forty-nine high-grade glioma patients in the test data set.

Next, we further explored which hub miRNAs can be effectively used as a prognosis signature for high-grade gliomas (III and IV). Likewise, high-grade glioma patients were divided into training and test subsets, and there were no significant difference in clinical characteristics (Supplementary Table S5). Then, a set of five miRNAs (hsa-miR-544, hsa-miR-628-5p, hsa-miR-139-5p, hsa-miR-524-5p and hsa-miR-15a) was identified to be correlated with patient survival in the training set (see details in Supplementary Table S6). Four of these five miRNAs were protective factors and the other one acted as a risky factor (Supplementary Table S6). Moreover, patients in high-risk group had a significantly shorter median survival than those with low-risk score in both training and test sets (Figures 4A and B). In the training set, the median survival time of high-risk group was 12.73 months and that of low-risk group was 18.55 months; on the other hand, the median survival time of high- and low-risk group in the test set was 15.40 months and 21.10 months, respectively. In addition, by analysing other known clinical prognostic factors for high-grade glioma, we found that besides the miRNA signature identified (*P* = 7.36e-6), *IDH1* mutation status was also associated with the survival of high-grade glioma patients in univariate analysis (*P* = 0.026). However, in multivariate analysis, only the signature comprising of five hub miRNAs was detected as an independent predictor (*P* = 0.005, detailed in Supplementary Table S7). These results suggest that these five hub miRNAs can effectively stratify high-grade glioma patients into different risk groups, and may play important roles in high-grade glioma.

### A hub miRNA signature predicts survival in GBMs

**Figure 5.**
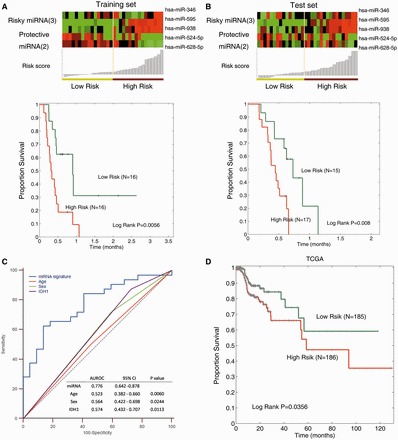
A five-miRNA signature effectively predicts survival of GBM patients. (**A**) Thirty-two GBM patients in the training data set. (**B**) Thirty-two GBM patients in the test data set. (**C**) Comparisons of the sensitivity and specificity for prediction of survival by the miRNA signature, age, sex and *IDH1* mutation status in GBM patients. The *P*-values show the area under the ROC (AUROC) of miRNA signature versus the AUROC of age, sex and *IDH1* mutation status. (**D**) Kaplan–Meier curve of overall survival according to the expression of the three miRNAs in the TCGA patients.

GBM is the most common and aggressive brain tumour with poor patient median survival. To explore whether these hub miRNAs can also predict GBM patient survival, we analysed the miRNA expression of GBM patients in our CGGA cohort. After randomly dividing GBM patients into training and test sets with equal number in each group (Supplementary Table S8), a five-miRNA signature was identified in the training set by univariate Cox regression analysis. Of these five miRNAs, hsa-miR-628-5p and hsa-miR-524-5p were found to be protective miRNAs and other three (hsa-miR-938, hsa-miR-595 and hsa-miR-346) were noted to act as risky factors (Supplementary Table S9). Then, the samples in training set were segregated into high- and low-risk groups, and these two groups exhibited significantly different survival times (Figure 5A, *P* = 0.0056); the median survival time of high-risk group was 9.68 months and that of low-risk group was 17.77 months. Moreover, the potential of the miRNA signature was independently validated in the test set (Figure 5B), and GBM patients with high-risk scores had overall poor survival, when compared with patients with low-risk scores (*P* = 0.008). In addition, in univariate Cox regression analysis, only the miRNA signature could independently predict the survival of GBM (*P* = 3.02e-7), and clinical (age and sex) and genetic variables (*IDH1* mutation status) were not identified as risky factors (Supplementary Table S10). Furthermore, we also used receiver operating characteristics (ROC) analysis to compare the sensitivity and specificity for the prediction of survival by the miRNA signature, age, sex and IDH mutation status. As a result, the five-miRNA signature also showed better prediction of survival than age, sex and *IDH1* mutation status with regard to overall survival in the combined GBM patients (Figure 5C). In addition, we also investigated whether the combination of these metrics with miRNA signature could achieve a better performance; however, the AUC was just comparable with that obtained based on the miRNA signature individually.

To reconfirm our miRNA signature as an independent predictor, we used the independent cohort of The Cancer Gene Atlas (TCGA), which provides the miRNA expression levels in a total of 371 GBMs. In the miRNA expression profiles obtained from TCGA, three of five miRNAs were assayed (hsa-miR-346, hsa-miR-524-5p and hsa-miR-595). To evaluate the performance of our miRNA signature, we first performed Z-score transformation on expression levels for each of the three miRNAs and then summarized the Z-scores of these three miRNAs as the risk score for each patient. Using the median value of these scores as the threshold, the GBM patients were divided into a high-risk group and a low-risk group. Likewise, Kaplan–Meier analysis indicated that our miRNA signature significantly classified the patients into a high-risk group and a low-risk group (Figure 5D, *P* = 0.0356).

### Network-based method increases the ability to identify the prognostic biomarkers

**Figure 6.**
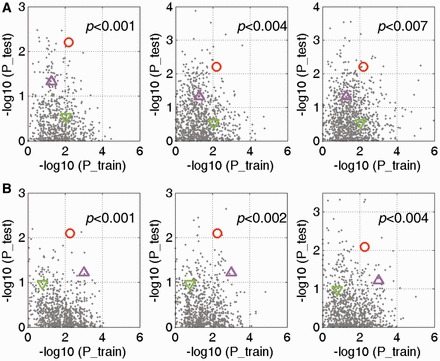
Network-based approach increases the ability to identify the miRNA prognostic signature. (**A**) Comparisons of miRNA signature prognostic ability in high-grade gliomas. Left panel, random miRNAs from non-hub miRNAs in the FMRN; middle panel, random miRNAs from differentially expressed miRNAs; right panel, random miRNAs from all miRNAs in the microarray. The green triangle indicates the most differentially expressed 21 miRNAs in type III, while the pink triangle denotes the most differentially expressed 21 miRNAs in GBMs. The red circle indicated the results of 21 hub miRNAs. (**B**) Comparisons of miRNA signature prognostic ability in GBMs.

Next, to assess whether the three hub miRNA signatures identified by our integrated approach are better than those detected by the traditional approach only based on miRNA expression, we first compared the prognostic performance of these hub miRNAs with most differentially expressed miRNAs detected in grade III glioma or GBMs. As control, 21 miRNAs with most differential expression were respectively chosen from these two differentially expressed miRNA lists, and then they were considered as the candidate miRNAs for our devised miRNA risk scoring system. Finally, their prognostic performances were evaluated in both training and test data sets by using the negative log-transformed *P* -values of log-rank tests. The higher the two scores, the better of the prognostic signature performances. For miRNAs with the most differential expression in grade III glioma, seven out of these miRNAs were identified to be significantly correlated with high-grade glioma patients’ survival in the training set. These seven-miRNA signature has no capacity to classify the test patients of high-grade glioma into different risk groups (log-rank *P* = 0.2938, Figure 6A, green downward triangle), although it could stratify the samples into different risk groups in the training data set (*P* = 0.0087). In addition, one miRNA (hsa-miR-384) was detected in the training set of GBM; however, it could not stratify GBMs either in the training or test data sets (*P* = 0.1632 and 0.1098 for the training and test sets respectively; Figure 6B, green downward triangle). On the other hand, for the miRNA set with the most differential expression in GBM, two miRNA signatures were respectively detected in the training sets comprising high-grade glioma or GBM patients. Only hsa-miR-524-5p was identified in the training set of high-grade glioma, which was also one of the hub miRNAs that we analysed. This miRNA exhibited moderate ability in both training and test data sets, relatively lower compared with the hub miRNA signature (Figure 6A, pink triangle). In addition, seven miRNAs, also including hsa-miR-524-5p, were detected in the training set of GBM; however, it cannot stratify GBMs in the test data sets (*P* = 0.0606, Figure 6B, pink triangle). Therefore, the prognostic performances of these hub miRNA signatures were better than those only considering differential expressions, especially in those test data sets. Thus, the network-based approach could increase the ability to identify the prognostic biomarkers.

Moreover, three comparisons were additionally performed to evaluate the prognostic performance of the miRNA signatures identified based on the FMRN, which were realized by three different randomization tests by randomly choosing miRNAs from three different backgrounds: non-hub miRNAs in the FMRN, all miRNAs differentially expressed in grade III and IV compared with grade II glioma and miRNAs assayed in microarray. As shown in Figure 6, the miRNA signatures based on the FMRN exhibited the best comprehensive performance both in training and test data sets in the context of high-grade glioma (Figure 6A, each subgraph corresponding to one kind of the three randomization tests) and GBMs (Figure 6B, each subgraph corresponding to one kind of the three randomization tests), and there was nearly no random signatures better than our network-based signature (red circles, *P* values < 0.005). We took these results as evidence that the network-based nature of the signature detection leads to the hub miRNA signatures with higher performance than non network-based methods.

### Functional roles of the hub miRNA signature associated with high-grade glioma

**Figure 7.**
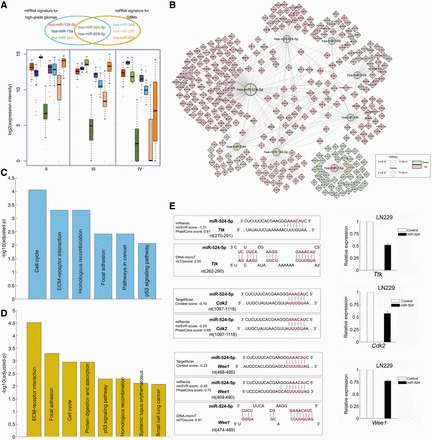
The functional roles of the miRNA signature in glioma. (**A**) The expression levels of the five miRNAs in three subtypes of glioma patients. The colour of the box plot corresponds to the font colour of miRNAs. (**B**) A subnetwork of the miRNA signatures and their targets abstracted from FMRN. (**C**) The KEGG pathways that are significantly enriched by targets of miRNA signatures in high-grade gliomas. (**D**) The KEGG pathways that are significantly enriched by targets of miRNA signatures in GBMs. (**E**) hsa-miR-524-5p regulates three targets, *Ttk*, *Cdk2* and *Wee1*. The detailed layout of the predicted regulatory information regarding hsa-miR-524-5p in three targets. Expressions of these three target genes are down-regulated by transfection of hsa-miR-524-5p in LN229 glioma cell.

In the above-mentioned section, a five-hub miRNA signature was found to be an independent predictor of high-grade glioma patient’s survival, in which four miRNAs (hsa-miR-524-5p, hsa-miR-628-5p, hsa-miR-139-5p and hsa-miR-544) were protective factors and the other one (hsa-miR-15a) acted as a risky factor. We further found that the expressions of four protective miRNAs were down-regulated not only in high-grade glioma and GBM compared with that in grade II glioma, but also in the progression from grade III glioma to GBM (Figure 7A), indicating that their high expressions would suppress the progression of glioma and might act as tumour suppressors. Indeed, in our previous studies, we had demonstrated that hsa-miR-524-5p was a brain-specific miRNA and was associated with the pathological grade and overall survival of gliomas (38). Consistent with the result of microarray, the relative expression of hsa-miR-524-5p in GBM cell lines was attenuated, when compared with low-grade glioma cell line H4. In addition, another three protective members were also found to be involved in glioma. Hsa-miR-139-5p had been investigated in more than five studies, which exhibited consistently down-regulation in GBM, and was found to be involved in the mesenchymal mode of migration and invasion, demonstrating the importance of miRNAs in the context of the cellular niche (39). However, its regulatory target genes have not yet been validated; hsa-miR-628-5p and hsa-miR-544 were also currently investigated. In GBMs, hsa-miR-628-5p showed down-expression (40), and hsa-miR-544 has been identified to exhibit a progression-associated down-regulation in glioma tumours, whose expression decreased significantly in anaplastic gliomas or GBM, when compared with low-grade gliomas (41). All these evidences support their protective roles in tumour progression. On the other hand, hsa-miR-15a, as a risky factor, was over-expressed in both high-grade glioma and GBM, and its expression in GBM was also higher than that in high-grade glioma, indicating that it might be an oncogenic miRNA. Previously, hsa-miR-15a has been found in the high-grade glioma, which is in accordance with our findings of its risky roles (42), and suppression of hsa-miR-15a has been noted to reduce the glioma cell migration ability (43). From these results and evidences, it can be concluded that the five-hub miRNA signature not only works well as the predictor of high-grade glioma patient’s survival, but also might play important functions in single or combination way in the process of glioma malignant progression.

Next, we further explored the putative biological functions of this five-miRNA prognostic signature via their regulatory target genes in the FMRN. We found that each of these five miRNAs regulates at least 37 targets, especially hsa-miR-524-5p controlling 142 progression-associated genes, which is also the one regulating the most genes in the FMRN. Moreover, we found that these miRNAs regulate many target genes in a combinational manner; in particular, hsa-miR-524-5p has the most combinational regulations with other three protective miRNAs (Figure 7B). In addition, we found that the expressions among four protective miRNAs in the high-grade glioma patients were significantly positively correlated, except for the pair of hsa-miR-544 and hsa-miR-139-5p (Supplementary Table S11). Meanwhile, hsa-miR-15a was significantly negatively associated with other four protective miRNAs (Supplementary Table S11). These results indicated that these miRNAs might have potential functional association. Functional enrichment analysis was also performed for target genes regulated by these five miRNAs, which showed that these genes were significantly enriched in many critical biological pathways, such as ‘cell cycle’, ‘extracellular matrixc (ECM)–receptor interaction’, ‘focal adhesion’ and ‘P53 signalling pathway,’ etc. (Figure 7C), revealing their potential important roles in direct tumourigenesis. Most of these functions are consistent with the left subnetwork of the FMRN. Among the 285 high-grade glioma-associated target genes, eight were annotated in KEGG cell cycle pathway, while 66 genes were annotated in ‘cell cycle’ term of gene ontology, 71.21% of which were targeted by hsa-miR-628-5p and hsa-miR-524-5p. There is increasing evidence that dysregulation of cell cycle genes and cell cycle regulatory genes are frequent events in human gliomas. Among these genes, *Ttk* was regulated by three of the five miRNAs (hsa-miR-139-5p, hsa-miR-524-5p and hsa-miR-628-5p), *Top2a* was regulated by hsa-miR-139-5p and hsa-miR-628-5p, and several other important cell cycle-related genes were regulated by hsa-miR-524-5p, including *Wee1*, *Cdk2* etc. *Ttk* has been demonstrated to play important roles in cell cycle and cell proliferation. It has been found to be a critical mitotic checkpoint protein for accurate segregation of chromosomes during mitosis. Tumourigenesis may occur when this protein fails to degrade and produces excess centrosomes resulting in aberrant mitotic spindles. *Cdk2* also plays important roles in cell cycle to regulate progression from G1 into S phase (44). Mir *et al.* have demonstrated that *Wee1* is a major regulator of the G2 checkpoint in GBM cells, and inhibition of *Wee1* by siRNA or small molecular compound in cells results in abrogation of the G2 arrest, premature termination of DNA repair and cell death (45). Its over-expression has been described previously for several types of cancer (46). We found that the expression of these genes increases with the progression of glioma (Supplementary Figure S2), suggesting their oncogenesis roles in the progression of gliomas. Another important function, ECM–receptor interaction, is thought to affect cell migration and also play critical roles in the progression of cancers. Six genes were enriched in this pathway, mainly including several collagen genes (*Col1a2*, *Col3a1* and *Col4a1*), 83.33% of which were targeted by hsa-miR-628-5p and hsa-miR-524-5p. During cancer progression, tumour cells eventually invade the surrounding collagen-rich extracellular matrix. Collagen is a major component of the interstitial extracellular matrix, and previous reports showed that some were dysregulated in various types of cancers. *Col4a1* has been shown to be over-expressed in glioma (47), and our current study showed that it was regulated by hsa-miR-524-5p and hsa-miR-628-5p. The down-regulation of two protective miRNAs may further increase the expression of *Col4a1* to promote the progression of glioma. In addition, consistent with previous studies, we also identified various genes regulated by these miRNAs involved in cell proliferation and cell apoptotic process.

Taken together, we have identified that these miRNAs might behave as key regulators by negatively targeting genes associated with cell cycle, cell proliferation, migration and invasion etc., playing extensive important roles in glioma malignant progression.

### Putative biological functions of the prognostic signature in GBM

For GBM, we also identified a signature composed of five hub miRNAs to predict survival, in which both hsa-miR-524-5p and hsa-miR-628-5p were also found to be protective factors and the other three miRNAs (hsa-miR-938, hsa-miR-595 and hsa-miR-346) were found to act as risky ones. As described earlier, hsa-miR-524-5p and hsa-miR-628-5p were the protective members of the signature of high-grade glioma. Here, we further found that these two miRNAs have the same roles in GBM, especially, the average expression of hsa-miR-524-5p in GBMs reduced to 8.40% of the expression level of type II samples. We further found that the expressions of the other three miRNAs were also gradually down-regulated following the progression of glioma (Figure 7A), and that they were novel disease miRNAs contributing to glioma malignant progression and were important members of the five-hub miRNA signature for GBM.

Next, we further explored the putative biological functions of this five miRNA prognostic signature via their regulatory target genes in the FMRN. Interestingly, hsa-miR-524-5p had combinational regulations with all other four miRNAs, and all these regulations formed a connected subnetwork (Figure 7B). Thus, the miRNA–mRNA regulatory subnetworks exhibited the regulatory multiplicity and cooperation of miRNAs. In addition, we found that the expression between hsa-miR-524-5p and hsa-miR-628-5p was significantly positively correlated in GBM (*R *= 0.41, *P* = 8.84e-4), meanwhile the other three risky miRNAs also exhibited expression correlations (Supplementary Table S12). Functional enrichment analysis was performed for target genes regulated by these five miRNAs, which also revealed that a number of these targets were implicated in pathways that have important roles in direct tumour growth and metastasis (Figures 7C and D). We previously found that restoring hsa-miR-524–5p expression in glioma suppressed cell proliferation, cell cycle and invasion both *in vitro* and *in vivo* (38). Therefore, hsa-miR-524-5p was a candidate GBM suppressor miRNA. In the current study, systematic analysis of the aberrantly expressed targets of hsa-miR-524-5p highlighted its critical role in cell cycle. Deregulation of the cell cycle machinery has been considered to be a factor in tumour generation; however, the cause of aberrantly high expression in GBM remains unclear. As described earlier, *Ttk*, *Cdk2* and *Wee1* were found to be critical members involved in cell cycle, and were negatively regulated by hsa-miR-524-5p as well (Figure 7B). Moreover, re-introduction of hsa-miR-524-5p in glioma cells showed that *Ttk*, *Cdk2* and *Wee1* expression were reduced in LN229 cells via transfecting hsa-miR-524-5p (Figure 7E).

## DISCUSSION

In this study, to improve our understanding of miRNAs on glioma malignant progression, a multiple-step method was proposed for constructing the FMRN by integrating paired miRNA and mRNA expression profiles and predicted miRNA–mRNA regulatory information, and signatures comprising hub miRNAs were further identified to be significantly associated with the survival of glioma patients. In the FMRN, not only miRNAs and genes are differentially expressed in both grade III and IV glioma, but the regulations between them are also associated with glioma progression. The identified edges might represent the direct regulations from miRNAs to mRNAs underlying the progression of glioma. When compared with all the regulations considered, only a small fraction showed aberrant behaviors in glioma progression, implying that although miRNAs have many different targets—dozens in some cases and hundreds in others—in the specific glioma cells, it seems that only a small number of them have a crucial role in cancer pathogenesis. Moreover, two separate subnetworks suggest two different functions of miRNAs involved in glioma progression. The miRNAs in the left subnetwork of the FMRN might mainly act as tumour suppressors, while those in the right subnetwork act as oncogenes. Furthermore, most miRNAs with the tumour-suppressive effects in glioma progression were newly detected, and their target genes highlight a significant influence on biological pathways directly relevant to neurodevelopment and tumourigenesis.

The FMRN was constructed by considering the differential expression of miRNAs and genes and active miRNA–target relationships in the progression of glioma. Active miRNA–target relationships are needed to characterize the functions of miRNAs in a specific context of disease, which can help in identifying the dysfunctional miRNAs associated with glioma progression. The miRNA signatures identified in our study not only have prognostic value in glioma, but also regulate more genes in the FMRN, indicating their important functional roles in tumourigenesis and progression. We found that some miRNAs were all identified in three miRNA signatures (Supplementary Figure S3), indicating their crucial roles in the progression of gliomas, such as hsa-miR-524-5p and hsa-miR-628-5p. Indeed, we found that the signatures identified in high-grade glioma and GBM are both involved in many tumour-related functions. More importantly, a striking proportion of target genes were implicated in cell cycle, and tended to be regulated by hsa-miR-524-5p and hsa-miR-628-5p. Typically, *Ttk*, *Cdk2* and *Wee1*, the critical members of cell cycle, were not only regulated by the miRNA signatures, but also relatively strictly regulated by miRNAs in the FMRN. Moreover, these target genes were all repressed by hsa-miR-524-5p in microarray analysis. In addition, their expressions were dominantly reduced after transfection of hsa-miR-524-5p. Here, we also found that hsa-miR-524-5p has multiple co-regulations with other members within the signatures, forming connected subnetworks. It has been noted that hsa-miR-524-5p is a brain-specific miRNA, and is associated with the pathological grade and overall survival of gliomas. These results provided models of the molecular mechanisms underlying complex diseases, and can broaden our understanding about the molecular regulatory mechanism of hsa-miR-524-5p. In addition, hsa-miR-628-5p and hsa-miR-15a are the other two examples related to glioma progression, which are worthy to be further studied. Thus, the miRNA signatures identified in our study could assist in the exploration of the functional mechanism behind the prognostic value of miRNAs in glioma, and might generate potential molecular targets for development of anticancer therapy.

Prognosis assessment is crucial for making appropriate treatment choices. In the present study, hub miRNAs were found to act as independent risk predictors to effectively distinguish the glioma patients with distinct survival, even in high-grade glioma and GBM. Their good performances were assessed in both training and test samples. In addition, when compared with the traditional method based on differentially expressed miRNAs to identify the prognostic signatures, our network-based method exhibited better performance. To investigate the influence of hub miRNA candidates on the performance of our proposed method, another two commonly used thresholds to define hubs (top 10 and 20%) were considered. As a result, we found that the network-based method also exhibited better performances than the traditional method (Supplementary Figure S4 and S5). In addition, in the process of identification of hub miRNAs, we also tried to consider the regulatory strength between a miRNA and its targets, and used the summation of the absolute correlation coefficients of expression levels as an alternative measure to weigh the central roles of miRNAs in the FMRN. The top 15% miRNAs with highest summary scores were the same as hub miRNAs based on degree in the text. Thus, our network-based method was robust in the identification of the miRNA prognostic signatures. In addition, although the *IDH1* gene has been discovered as a predictor of glioma survival in previous studies (48–50), it could not distinguish GBM patients in our Chinese populations (Supplementary Figure S6), suggesting the underlying genetic heterogeneity among different populations. Fortunately, a five-miRNA signature was effective to predict the survival in the cohort of Chinese GBM patients. Moreover, the miRNA signature still showed a better prediction of survival in the non-Asian patients. However, additional independent samples will be needed to further confirm our findings before the miRNA signatures identified in the present can be used in clinic globally.

Many efforts have been made to explore the molecular mechanism of GBMs, such as the REMBRANDT (51) and TCGA (52) projects, and many miRNAs were identified to be associated with GBMs or subtypes of GBMs. Here, we present a study of mRNA and miRNA profiles from 160 Chinese glioma patients with different grades. The data presented bridges the gap left by REMBRANDT and TCGA, and as such, its availability will be of great interest to the community. Meg Duroux and colleagues (39) performed a systematical review and aimed to present a comprehensive overview of all the available literature on the expression profiles and functions of miRNA in GBM, and totally, 365 miRNAs showed expression changes in GBM tumour samples, when compared with normal brain tissue. However, 85% of these miRNAs have not yet been functionally characterized. In the present study, we found that 96 out of 145 miRNAs in the FMRN were reported in the review (Supplementary Figure S7). This result indicated that a large proportion of miRNAs involved in glioma progression might also play important roles in GBMs. Next, we particularly analysed the three signatures identified in our study. First, for the 21 hub miRNAs composed of the prognosis signature for glioma, 66.67% were reported to be expression changes in GBMs. Here, our analysis suggested that they are also associated with the progression of glioma, extending our understanding about their functions in glioma. Second, all the five hub members of the miRNA signature for high-grade glioma were reported in previous studies; especially, hsa-miR-544 has been identified to exhibit a progression-associated down-regulation in glioma tumours, whose expression decreased significantly in anaplastic gliomas or GBM, when compared with low-grade gliomas. Another example was hsa-miR-139-5p, exhibiting consistently down-regulated in GBM, which was found to be involved in the mesenchymal mode of migration and invasion, demonstrating the importance of miRNAs in the context of the cellular niche. Finally, for the five hub miRNAs of the GBM signature, hsa-miR-524-5p and hsa-miR-628-5p were previously reported. In addition, when compared with the results reported by Mark D. Johnson and colleagues (53), we found 34 progression-associated miRNAs in the FMRN were identified as potentially markers of GBM subtypes, extending their important roles in GBMs. Therefore, majority of miRNAs involved in glioma progression are known GBM miRNAs, and this finding is in good agreement with the results presented in previous publications. Besides miRNAs working in GBM, a number of novel miRNAs associated with glioma progression have also been identified, especially, including some miRNAs with suppressive progression potential, providing the candidates for further studies in glioma.

Although there are a couple of miRNAs that were previously reported to play important roles in GBMs, we found that there are also several miRNAs not differentially expressed in the progression of glioma. This result might suggest that they were not involved in the malignant progression of glioma, or be explained by the differential experiment designs and the underlying genetic heterogeneity among different populations. For example, 4 out of 10 miRNA signature detected in GBMs by Srinivasan *et al.* (9) were differentially expressed in the progression of gliomas, and only one regulating 49 genes in the FMRN was one of the hub miRNAs. This miRNA was found to be a member of the miRNA signature in glioma; however, it was not associated with the overall survival of high-grade glioma and GBM. In addition, our previous studies have reported some miRNAs in GBMs, and part of them are also associated with the progression of glioma, such as hsa-miR-139-5p (54) and hsa-miR-524-5p (38).

In this study, we focused on the identification of the miRNA signatures associated with the glioma progression by considering their topological central roles in the FMRN. Similarly, ‘in-degree’ for genes also could measure their central roles in the FMRN. By computing the correlation between in-degree of gene in the FMRN and the negative log-transformed *P* value of survival analysis, in-degree is significantly positively associated with the power for survival analysis. These results suggest that the in-degree may be a good indicator for identifying the genes involved in the progression of gliomas, extending the application of the integrative method.

In summary, by integrative analyses, we not only identified the miRNA predictors of survival, but also analysed the functional roles of miRNAs in the context of glioma progression. In conclusion, systematic analysis of the FMRN provides new insights into post-transcriptional gene regulation in the progression of glioma.

## SUPPLEMENTARY DATA

Supplementary Data are available at NAR Online, including [55–57].

## FUNDING

The Innovation Research Fund for Graduate Students of Heilongjiang province [YJSCX2012-196HLJ]. Funding for open access charge: Funds for Creative Research Groups of The National Natural Science Foundation of China [81121003]; National Natural Science Foundation of China [91129710, 61170154, 61203264]; Specialized Research Fund for the Doctoral Program of Higher Education of China [20102307110022]; China Postdoctoral Science Foundation [2012M520764]; Heilongjiang Postdoctoral Fund [LBH-Z12214]; The Innovation Research Fund for Graduate Students of Heilongjiang province [YJSCX2012-196HLJ].

*Conflict of interest statement*. None declared.

## Supplementary Material

Supplementary Data
