# Supplementary material for: Comprehensive analysis of the functional microRNA–mRNA regulatory network identifies miRNA signatures associated with glioma malignant progression
Source: Nucleic Acids Res. 2013 Nov 3;41(22):e203. doi: 10.1093/nar/gkt1054 (PMC3905890; doi:10.1093/nar/gkt1054)
Supplement: Supplementary Data [file supp_41_22_e203__index.html]

Comprehensive analysis of the functional microRNA–mRNA regulatory network identifies miRNA signatures associated with glioma malignant progression — Comprehensive analysis of the functional microRNA–mRNA regulatory network identifies miRNA signatures associated with glioma malignant progression — Supplementary Data 

# Comprehensive analysis of the functional microRNA–mRNA regulatory network identifies miRNA signatures associated with glioma malignant progression

## Supplementary Data

files

**Files in this Data Supplement:**

- Supplementary Data - pdf file
- Supplementary Data - xls file
