# Supplementary material for: Comprehensive analysis of the functional microRNA–mRNA regulatory network identifies miRNA signatures associated with glioma malignant progression
Source: Nucleic Acids Res. 2013 Nov 3;41(22):e203. doi: 10.1093/nar/gkt1054 (PMC3905890; doi:10.1093/nar/gkt1054)
Supplement: Supplementary Data [file supp_gkt1054_nar-02547-met-g-2013-File009.pdf]

Comprehensive analysis of the functional microRNA-mRNA regulatory network identifies miRNA signatures associated with glioma malignant progression

Yongsheng Li[1], Juan Xu[1], Hong Chen[1], Jing Bai[1], Shengli Li[1], Zheng Zhao[1], Tingting Shao[1], Tao Jiang[2], Huan Ren[3,*], Chunsheng Kang[4,*], and Xia Li[1,*]

Supplementary Materials and Methods......3
Patient characteristics......3
RNA extraction, mRNA and microRNA microarrays......3
Comprehensive list of in-silico predicted miRNA targets......4
Cell culture and transfection......4
References......5
Supplementary Tables and Figures......6
Supplementary Table S1. Clinicopathologic characteristics of patients with glioma in the CGGA cohort (n = 160). 6
Supplementary Table S2. The functional miRNA-mRNA regulatory network......8
Supplementary Table S3. Univariate Cox regression analysis of the 21 hub microRNAs associated with overall survival in the training set of 80 glioma patients. 8
Supplementary Table S4. Cox regression analysis of risk score obtained by 21 hub miRNAs and age in the combined glioma patient set (n = 160). 9
Supplementary Table S5. Clinicopathologic characteristics of patients with high-grade glioma in the CGGA cohort (n = 97)......10
Supplementary Table S6. Univariate Cox regression analysis of the 21 hub microRNAs associated with overall survival in the training set of 48 high-grade glioma patients......11
Supplementary Table S7. Cox regression analysis of risk score obtained by 21 hub miRNAs and age in the combined high-grade glioma patient set (n = 97). 12
Supplementary Table S8. Clinicopathologic characteristics of patients with GBM in the CGGA cohort (n = 64). 13
Supplementary Table S9. Univariate Cox regression analysis of the 21 hub microRNAs associated with overall survival in the training set of 32 GBM patients. 14
Supplementary Table S10. Univariate Cox regression analysis of the five miRNA signature and clinical variables associated with overall survival in the combined set of 64 GBM patients. 15
Supplementary Table S11. The correlation of five miRNAs in the signature for high-grade glioma patients. 16
Supplementary Table S12. The correlation of five miRNAs in the signature for GBM patients. ...17
Supplementary Figure S1. Overview of the integrative approach for construction of the functional miRNA-mRNA regulatory network and miRNA signatures identification. 18
Supplementary Figure S2. The expression levels of targets of miRNA signatures......19
Supplementary Figure S3. A Venn diagram to illustrate the three miRNA signatures identified in the study. 20
Supplementary Figure S4. Network-based approach increases the power to identify the miRNA

prognostic signature (top 10% as hubs). 21
Supplementary Figure S5. Network-based approach increases the power to identify the miRNA prognostic signature (top 20% as hubs). 22
Supplementary Figure S6. Kaplan-Meier curve of overall survival according to the IDH1 mutation status in the GBM patients. 23
Supplementary Figure S7. Most of the miRNAs in the FMRN were reported to be associated with glioma in previous studies. 24

Supplementary Materials and Methods

Patient characteristics

Biopsy tissue samples of glioma tumors and patients' clinical pathologic information were received from Beijing Tiantan hospital. Written informed consent was obtained from all donors. Ethical approval for analysis of samples and notes was obtained from the local research ethics committee. All these samples were histologically graded according to current WHO classification of tumors of the nervous systems. In total, 160 samples from patients were analyzed in this study, including 63 WHO grade II patients, 33 grade III patients and 64 GBM patients.

RNA extraction, mRNA and microRNA microarrays

All tissue samples were immediately snap-frozen in liquid nitrogen after surgery. A hematoxylin and eosin-stained frozen section was prepared from each glioma sample to assess the percentage of tumor cells before RNA extraction. Only samples that contained >80% tumor cells were selected. Total RNA from frozen tumor samples was extracted using the mirVana miRNA Isolation kit (Ambion) according to the manufacturer's protocol. RNA concentration and quality were measured using the NanoDrop ND-1000 spectrophotometer (NanoDrop Technologies). The gene expression profiling was performed using the Agilent Whole Human Genome Array according to the manufacturer's instructions. The integrity of total RNA was checked using an Agilent 2100 Bioanalyzer (Agilent). cDNA and biotinylated cRNA were synthesized and hybridized to the array. Data were acquired using the Agilent G2565BA Microarray Scanner System and Agilent Feature Extraction Software (version 9.1). In addition, the miRNA expression profiling was performed using the human v2.0 miRNA expression BeadChip (Illumina, Inc. San Diego, Calif) according to the manufacturer's instructions. The data were background subtracted and normalized using quantile normalization with Illumina BeadStudio software v3.1. Then log transformation (base 2) was performed.

All data were deposited in Chinese Glioma Genome Atlas (CGGA) and the sample IDs, survival times were provided in Supplementary Table S1. We collected gene expression

profiling from the CGGA, which includes a total of 220 glioma samples and has been used to validate a new molecular subtyping system of GBM (55). In this study, we only focused on mRNA expression profiling of the 160 glioma samples that were also measured miRNA expression, which has also been uploaded to the CGGA. Table 1 summarizes the information of WHO grade of glioma, patient ages and sexes, and complete clinical follow-up data.

Comprehensive list of in-silico predicted miRNA targets

An extremely small number of miRNA targets have been validated previously (56), thus analyses of miRNA-target regulations rely on in silico predictions. Although several algorithms have been suggested for target prediction, results from these algorithms present significant discrepancies (57). Accurate target prediction is still challenging. Discrepancies are mainly due to differences in implementation, different requirements for site conservation across species and different hypotheses regarding the miRNA action on its target genes. Algorithms used in this study were: TargetScan5.1 (20), miRanda (miRBase version5) (19), Pictar (four-way) (21) and DIANA-microT (version 3.0) (22). We directly downloaded the human miRNA targets from these databases. Entrez gene IDs were used as common identifiers.

In this study, we build on previous results and in particular on the conclusion common in multiple studies that using the union of the predictions from these algorithms rather than the intersection provides a more promising strategy to select true targets (23,24). In addition, we expected that a gene was thought to be regulated by a miRNA, only if it has the binding sites of the miRNA and its expression is inversely correlated with the miRNA expression. Integrating the predicted target information with the expression, we can get the true miRNA targets functions in the context of progression of glioma.

Cell culture and transfection

Human LN229 glioblastoma cells were obtained from the China Academia Sinica Cell Repository, Shanghai, China. The cells were maintained in Dulbecco’s modified Eagle’s medium (Gibco, Los Angeles, CA) supplemented with 10% fetal bovine serum (Gibco), and were incubated at $37^{o}C$ in a 5% CO2 atmosphere. Hsa-miR-524-5p mimics were chemically

synthesized and purified by high-performance liquid chromatography (GenePharma, Shanghai, China). Cells were transfected with miR-524-5p mimics (200 pmol) using Lipofectamine 2000 (Invitrogen). Cells transfected with scrambled 2'-OMe oligonucleotides (scramble) were used as control. RNAs were isolated from the cells and then microarray was used to assay the expression levels of genes. In one of our previous study (38), we have shown restored expression of miR-524-5p in glioma suppressed cell proliferation and invasion both in vitro and in vivo. Transient transfection of miR-524-5p mimics led to overexpression of miR-524-5p as determined by real-time PCR, indicating the transfection efficiency is effective (38).

References

55. Yan, W., Zhang, W., You, G., Zhang, J., Han, L., Bao, Z., Wang, Y., Liu, Y., Jiang, C., Kang, C. *et al.* (2012) Molecular classification of gliomas based on whole genome gene expression: a systematic report of 225 samples from the Chinese Glioma Cooperative Group. *Neuro Oncol*, **14**, 1432-1440.
56. Hsu, S.D., Lin, F.M., Wu, W.Y., Liang, C., Huang, W.C., Chan, W.L., Tsai, W.T., Chen, G.Z., Lee, C.J., Chiu, C.M. *et al.* (2011) miRTarBase: a database curates experimentally validated microRNA-target interactions. *Nucleic acids research*, **39**, D163-169.
57. Witkos, T.M., Koscianska, E. and Krzyzosiak, W.J. (2011) Practical Aspects of microRNA Target Prediction. *Curr Mol Med*, **11**, 93-109.

Supplementary Tables and Figures

Supplementary Table S1. Clinicopathologic characteristics of patients with glioma in the CGGA cohort (n = 160).

ID	OS(days)	ID	OS(days)	ID	OS(days)	ID	OS(days)
253	1658	595	825	393	463	311	230
258	1163	601	818	403	1165	324	336
275	1221	626	784	405	497	335	385
281	1235	633	761	406	290	342	526
285	1225	639	752	412	280	345	1024
316	1181	648	754	438	777	346	104
317	1180	659	725	474	1104	366	255
321	1173	663	721	476	1089	370	338
323	1172	672	711	489	653	371	811
357	1132	673	437	490	1097	373	281
365	260	688	692	492	652	375	657
379	1091	692	689	498	356	377	399
387	1084	708	663	508	285	380	165
396	376	711	662	513	1062	401	168
399	1063	712	661	558	613	419	198
402	1061	718	655	562	290	436	568
407	1054	736	629	596	962	437	694
433	1013	743	622	598	961	439	565
434	1012	746	619	11	155	442	559
446	997	753	613	104	162	444	225
447	1007	767	598	124	414	451	558
459	977	770	594	126	796	454	412
461	991	776	587	156	179	462	364
469	978	792	557	168	1471	464	403
484	941	231	145	172	462	504	563
485	937	232	429	178	966	512	588
505	923	249	312	195	455	518	204
522	908	259	549	203	188	527	439
542	887	260	567	205	292	547	580
543	867	314	1341	210	300	549	413
544	882	329	419	218	313	557	257
548	875	331	1304	221	287	570	561
552	873	334	1299	225	1407	573	554
571	854	351	742	240	386	575	554
579	831	352	1291	255	591	588	533

583	840	353	530	264	383	593	243
589	816	354	145	287	563	594	532
590	830	364	878	292	826	597	525
592	826	391	1234	308	810	604	381
606	325	609	512	612	504	822	593

Note: Other information of samples can be obtained in CGGA by sample ids.

Supplementary Table S2. The functional miRNA-mRNA regulatory network.

Supplementary Table S3. Univariate Cox regression analysis of the 21 hub microRNAs associated with overall survival in the training set of 80 glioma patients.

**microRNAs**	**HR (95%CI)**	**Regression coefficient**	**P-value***	**Type#**
**hsa-miR-1246**	2.523 (1.645-3.868)	0.925	**1.49e-5**	R
**hsa-miR-1301**	0.437 (0.292-0.654)	-0.827	**3.85e-5**	P
**hsa-miR-139-3p**	0.670 (0.542-0.828)	-0.400	**1.58e-4**	P
**hsa-miR-139-5p**	0.509 (0.355-0.730)	-0.674	**1.77e-4**	P
**hsa-miR-142-5p**	1.477 (1.238-1.762)	0.390	**1.02e-5**	R
**hsa-miR-148a**	2.191 (1.444-3.325)	0.784	**1.69e-4**	R
**hsa-miR-15a**	6.203 (2.786-13.814)	1.825	**5.13e-6**	R
**hsa-miR-302d**	0.763 (0.649-0.896)	-0.271	**7.75e-4**	P
**hsa-miR-339-5p**	2.083 (1.508-2.879)	0.734	**5.67e-6**	R
**hsa-miR-346**	0.651 (0.515-0.824)	-0.429	**2.65e-4**	P
**hsa-miR-504**	0.698 (0.567-0.859)	-0.360	**5.21e-4**	P
**hsa-miR-524-5p**	0.744 (0.657-0.841)	-0.296	**1.58e-6**	P
**hsa-miR-544**	0.867 (0.817-0.920)	-0.143	**1.42e-6**	P
**hsa-miR-548f**	0.576 (0.397-0.838)	-0.551	**3.21e-3**	P
**hsa-miR-586**	0.834 (0.734-0.949)	-0.181	**4.90e-3**	P
**hsa-miR-590-3p**	1.254 (1.087-1.447)	0.226	**1.52e-3**	R
**hsa-miR-595**	0.892 (0.830-0.960)	-0.114	**1.87e-3**	P
**hsa-miR-600**	0.836 (0.746-0.938)	-0.179	**1.81e-3**	P
**hsa-miR-628-5p**	0.531 (0.404-0.697)	-0.633	**3.35e-6**	P
**hsa-miR-924**	0.829 (0.735-0.934)	-0.188	**0.0017**	P
**hsa-miR-938**	0.935 (0.880-0.993)	-0.067	**0.0264**	P

*We calculated the hazard ratios (HRs) and p values with the unadjusted Cox proportional-hazards model in Matlab. The miRNAs in bold were the members of miRNA signature.

#P represents protective miRNAs while R represents risky miRNAs.

Supplementary Table S4. Cox regression analysis of risk score obtained by 21 hub miRNAs and age in the combined glioma patient set (n = 160).

	**Univariate analysis**			**Multivariate analysis**		
**Variable**	HR (95%CI)	Regression coefficient	P-value*	HR (95%CI)	Regression coefficient	P-value*
Age	1.038 (1.016-1.060)	0.037	**4.89e-4**	1.017 (0.995-1.039)	0.016	0.129
Sex	1.163 (0.707-1.914)	0.151	0.544			
IDH1	0.224 (0.119-0.418)	-1.498	**1.73e-6**	0.522 (0.259-1.052)	-0.651	0.063
miRNA signature	1.097 (1.068-1.127)	0.093	**5.64e-12**	1.076 (1.044-1.109)	0.074	**1.12e-6**

Supplementary Table S5. Clinicopathologic characteristics of patients with high-grade glioma in the CGGA cohort (n = 97).

Characteristics	Number of patients		P
	Training set N=48	Test set N=49	
Stage			0.99 [a]
III	16	17	
IV	32	32	
Sex			0.68 [a]
Female	17	20	
Male	31	29	
Age			0.28 [b]
Mean±SD	45.23±13.89	42.27±12.74	
Range	12-70	17-65	
IDH1 mutation			0.32 [a]
Mutated	8	14	
Wild	32	30	
Survival (month)			0.65 [b]
Mean±SD	18.13±11.88	19.16±10.14	
Range	3.47-49.03	4.83-43.47	
State			0.52 [a]
Living	30	34	
Death	18	15	

Abbreviations: IDH1, isocitrate dehydrogenase 1; SD, standard deviation.
[a] P values were determined using Fisher's exact test.
[b] P values were determined using student's t test.

Supplementary Table S6. Univariate Cox regression analysis of the 21 hub microRNAs associated with overall survival in the training set of 48 high-grade glioma patients.

microRNAs	HR (95%CI)	Regression coefficient	P-value*	Type#
hsa-miR-1246	1.600 (0.987-2.594)	0.470	0.052	
hsa-miR-1301	1.029 (0.580-1.823)	0.028	0.921	
hsa-miR-139-3p	0.832 (0.661-1.047)	-0.184	0.109	
**hsa-miR-139-5p**	0.572 (0.371-0.882)	-0.559	**0.010**	P
hsa-miR-142-5p	1.120 (0.872-1.437)	0.113	0.365	
hsa-miR-148a	1.247 (0.691-2.251)	0.221	0.455	
**hsa-miR-15a**	3.093 (1.154-8.288)	1.129	**0.022**	R
hsa-miR-302d	1.016 (0.845-1.222)	0.016	0.863	
hsa-miR-339-5p	1.358 (0.937-1.967)	0.306	0.099	
hsa-miR-346	1.077 (0.762-1.522)	0.074	0.669	
hsa-miR-504	0.963 (0.754-1.231)	-0.037	0.760	
**hsa-miR-524-5p**	0.852 (0.738-0.984)	-0.160	**0.027**	P
**hsa-miR-544**	0.927 (0.867-0.991)	-0.076	**0.024**	P
hsa-miR-548f	1.206 (0.695-2.091)	0.187	0.497	
hsa-miR-586	0.973 (0.813-1.165)	-0.027	0.762	
hsa-miR-590-3p	0.882 (0.737-1.055)	-0.126	0.161	
hsa-miR-595	1.005 (0.925-1.092)	0.005	0.898	
hsa-miR-600	1.030 (0.905-1.173)	0.030	0.645	
**hsa-miR-628-5p**	0.616 (0.439-0.866)	-0.484	**0.004**	P
hsa-miR-924	1.039 (0.870-1.241)	0.039	0.664	
hsa-miR-938	1.022 (0.958-1.091)	0.022	0.499	

*We calculated the hazard ratios (HRs) and p values with the unadjusted Cox proportional-hazards model in Matlab. The miRNAs in bold were the members of miRNA signature.

#P represents protective miRNAs while R represents risky miRNAs.

Supplementary Table S7. Cox regression analysis of risk score obtained by 21 hub miRNAs and age in the combined high-grade glioma patient set (n = 97).

	**Univariate analysis**			**Multivariate analysis**		
**Variable**	HR (95%CI)	Regression coefficient	P-value*	HR (95%CI)	Regression coefficient	P-value*
Age	1.017 (0.996-1.038)	0.016	0.111			
Sex	1.226 (0.730-2.057)	0.204	0.432			
IDH1	0.474 (0.243-0.927)	-0.746	**0.026**	0.775 (0.369-1.627)	-0.254	0.492
miRNA signature	1.493 (1.249-1.786)	0.401	**7.36e-6**	1.403 (1.102-1.786)	0.339	**0.005**

Supplementary Table S8. Clinicopathologic characteristics of patients with GBM in the CGGA cohort (n = 64).

Characteristics	Number of patients		P
	**Training set** N=32	**Test set** N=32	
Sex			0.79[a]
Female	10	12	
Male	22	20	
Age			0.44 [b]
Mean±SD	46±13.73	43.47±12.45	
Range	12-70	17-65	
IDH1 mutation			0.73 [a]
Mutated	4	6	
Wild	22	22	
Survival (month)			0.75[b]
Mean±SD	16.23±10.95	15.53+6.36	
Range	3.47-49.03	5.40-34.13	
State			0.99 [a]
Living	23	24	
Death	9	8	

Abbreviations: IDH1, isocitrate dehydrogenase 1; SD, standard deviation.

[a] P values were determined using Fisher's exact test.

[b] P values were determined using student's t test.

Supplementary Table S9. Univariate Cox regression analysis of the 21 hub microRNAs associated with overall survival in the training set of 32 GBM patients.

microRNAs	HR (95%CI)	Regression coefficient	P-value*	Type#
hsa-miR-1246	1.232 (0.703-2.160)	0.209	0.456	
hsa-miR-1301	0.913 (0.424-1.968)	-0.091	0.812	
hsa-miR-139-3p	0.904 (0.707-1.155)	-0.101	0.409	
hsa-miR-139-5p	0.718 (0.448-1.151)	-0.331	0.161	
hsa-miR-142-5p	0.950 (0.691-1.308)	-0.051	0.750	
hsa-miR-148a	1.030 (0.567-1.869)	0.029	0.921	
hsa-miR-15a	4.196 (0.707-24.894)	1.434	0.107	
hsa-miR-302d	1.225 (0.990-1.517)	0.203	0.057	
hsa-miR-339-5p	0.996 (0.556-1.785)	-0.004	0.990	
**hsa-miR-346**	1.631 (1.043-2.552)	0.489	**0.029**	R
hsa-miR-504	1.274 (0.934-1.739)	0.243	0.119	
**hsa-miR-524-5p**	0.849 (0.721-0.999)	-0.164	**0.044**	P
hsa-miR-544	0.956 (0.885-1.033)	-0.045	0.243	
hsa-miR-548f	1.653 (0.865-3.158)	0.503	0.120	
hsa-miR-586	0.980 (0.815-1.178)	-0.021	0.822	
hsa-miR-590-3p	0.883 (0.684-1.138)	-0.125	0.326	
**hsa-miR-595**	1.200 (1.059-1.360)	0.183	**0.004**	R
hsa-miR-600	1.083 (0.950-1.236)	0.080	0.223	
**hsa-miR-628-5p**	0.679 (0.470-0.982)	-0.387	**0.036**	P
hsa-miR-924	1.108 (0.918-1.337)	0.102	0.277	
**hsa-miR-938**	1.113 (1.029-1.204)	0.107	**0.007**	R

*We calculated the hazard ratios (HRs) and p values with the unadjusted Cox proportional-hazards model in Matlab. The miRNAs in bold were the members of miRNA signature.
#P represents protective miRNAs while R represents risky miRNAs.

Supplementary Table S10. Univariate Cox regression analysis of the five miRNA signature and clinical variables associated with overall survival in the combined set of 64 GBM patients.

	**Univariate analysis**		
**Variable**	HR (95%CI)	Regression coefficient	P-value*
Age	1.018 (0.992-1.044)	0.018	0.165
Sex	1.144 (0.615-2.126)	0.134	0.665
IDH1	0.520 (0.198-1.361)	-0.655	0.174
miRNA signature	1.579 (1.321-1.888)	0.457	**3.02e-7**

Supplementary Table S11. The correlation of five miRNAs in the signature for high-grade glioma patients.

P \ R	miR-544	miR-628-5p	miR-139-5p	miR-524-5p	miR-15a
miR-544	---	0.62	0.17	0.24	-0.54
miR-628-5p	8.37e-12	---	0.61	0.46	-0.59
miR-139-5p	0.09	3.14e-11	---	0.26	-0.38
miR-524-5p	0.02	1.94e-6	0.01	---	-0.41
miR-15a	1.56e-8	2.21e-10	1.00e-4	3.58e-5	---

Note: the upper triangular shows the Pearson correlation coefficient while the lower triangular shows the p-values.

Supplementary Table S12. The correlation of five miRNAs in the signature for GBM patients.

P \ R	miR-628-5p	miR-524-5p	miR-938	miR-595	miR-346
miR-628-5p	---	0.41	-0.40	-0.53	-0.20
miR-524-5p	8.84e-4	---	-0.07	-0.18	-0.03
miR-938	9.74e-4	0.57	---	0.73	0.21
miR-595	5.87e-6	0.15	5.69e-12	---	0.32
miR-346	0.11	0.81	0.10	9.55e-3	---

Note: the upper triangular shows the Pearson correlation coefficient while the lower triangular shows the p-values.

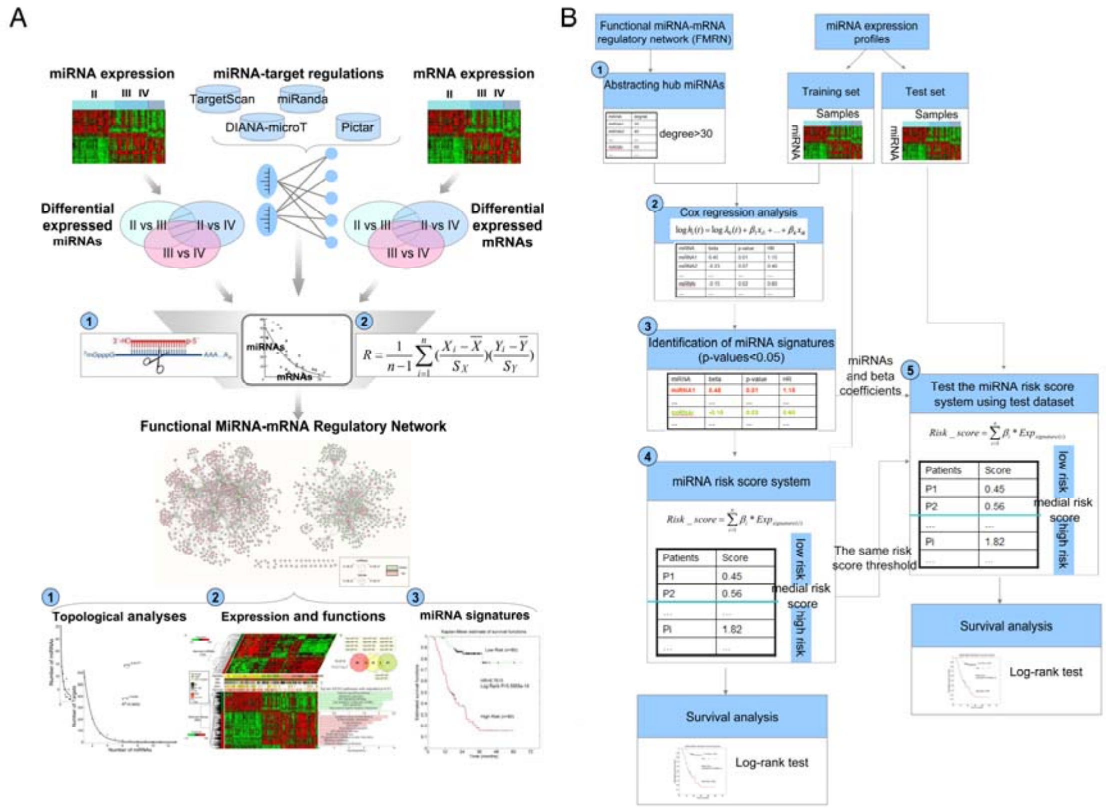

Supplementary Figure S1. Overview of the integrative approach for construction of the functional miRNA-mRNA regulatory network and miRNA signatures identification.

(A) The flowchart for construction of the FMRN. (B) The workflow for identification of the miRNA signatures.

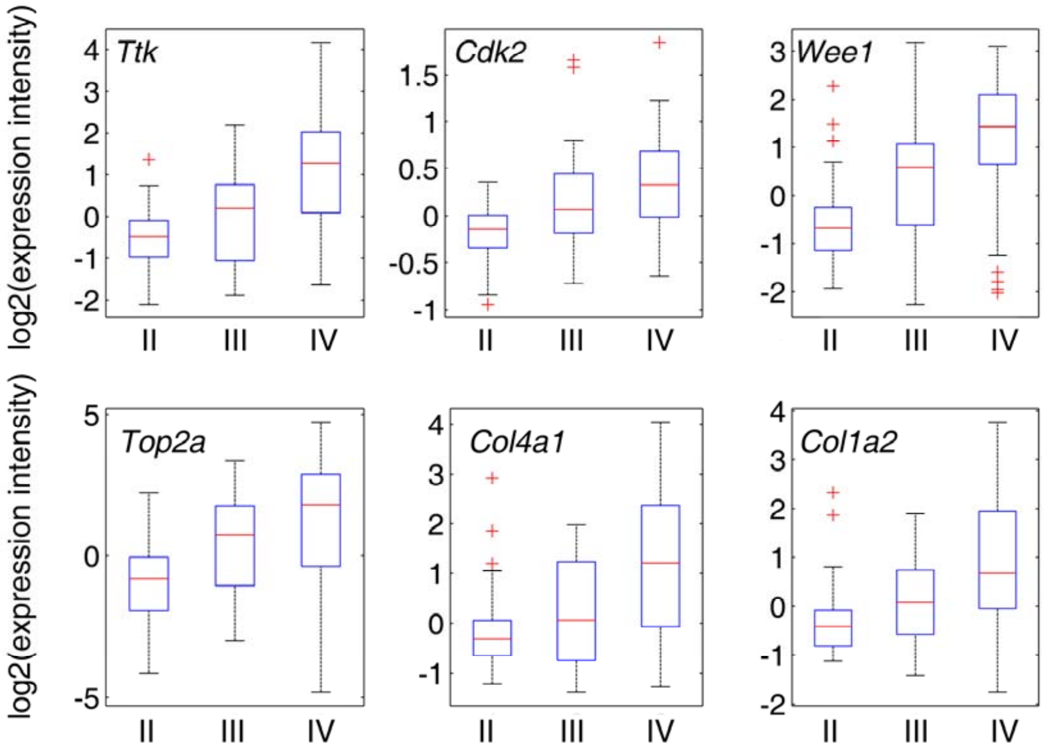

Supplementary Figure S2. The expression levels of targets of miRNA signatures.

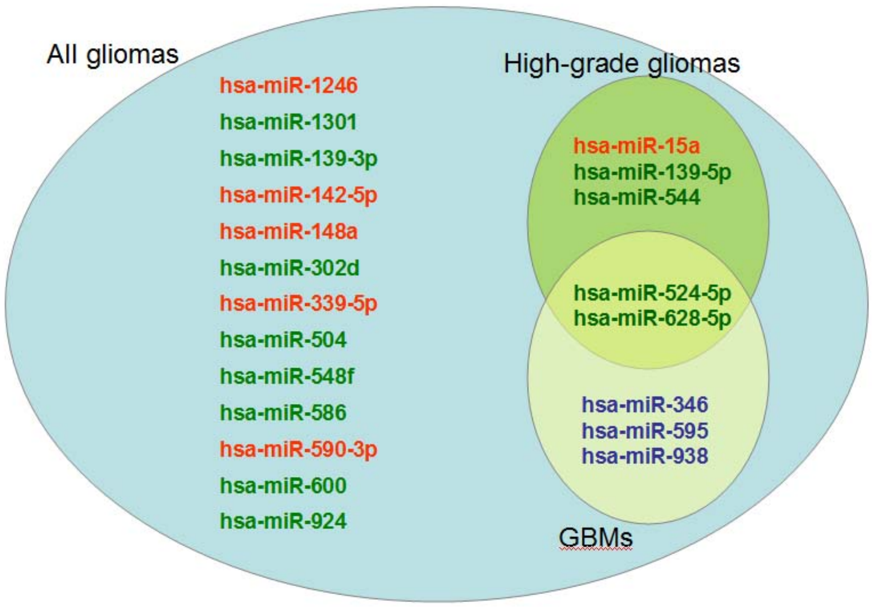

Supplementary Figure S3. A Venn diagram to illustrate the three miRNA signatures identified in the study.

Blue oval represents the miRNA signature for all gliomas, green oval represents the miRNA signature for high-grade gliomas and the light yellow one represents the miRNA signature for GBMs. The miRNAs with risky roles were colored red while the protective ones were colored green. The miRNAs with protective roles in all gliomas and risky roles in GBMs were colored blue.

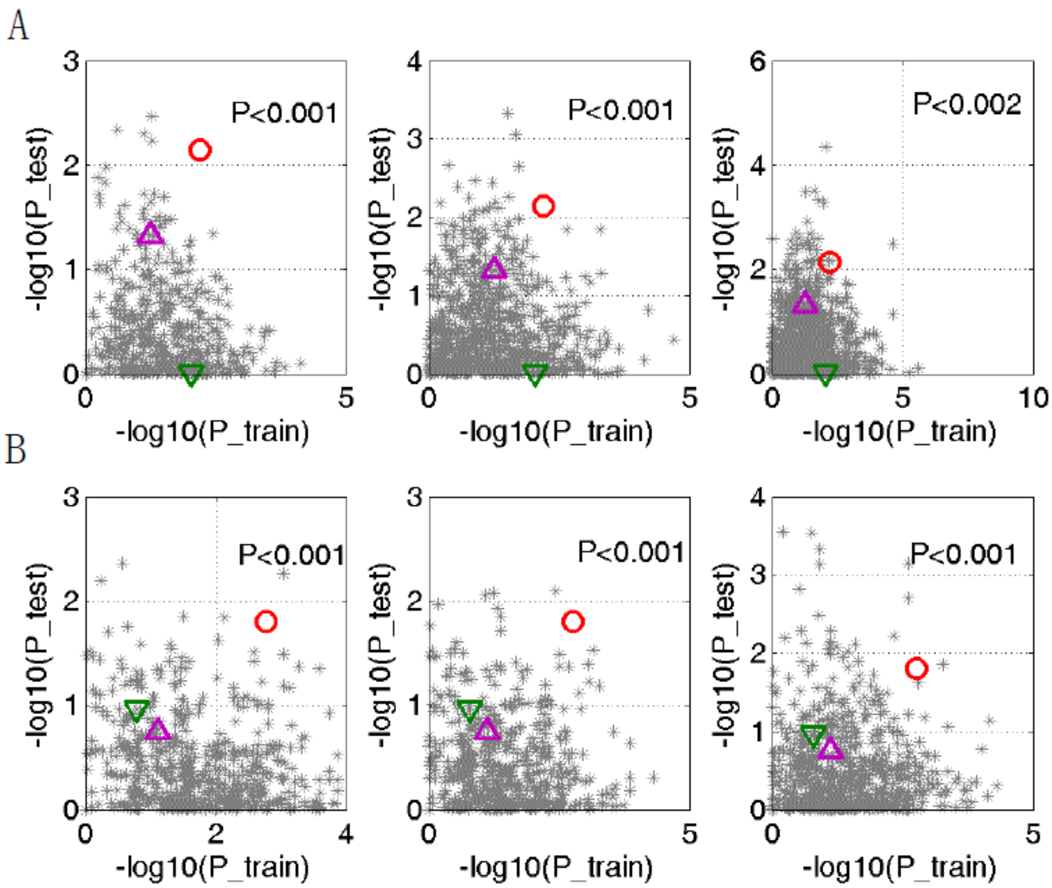

Supplementary Figure S4. Network-based approach increases the power to identify the miRNA prognostic signature (top 10% as hubs).

(A) Comparisons of miRNA signature prognostic power in high-grade gliomas. Left panel, random miRNAs from non-hub miRNAs in the FMRN; middle panel, random miRNAs from differentially expressed miRNAs; right panel, random miRNAs from all miRNAs in the microarray. The green triangle is based on the most differentially expressed 14 miRNAs in type III, while the pink triangle is based on the most differentially expressed 14 miRNAs in GBMs. The red circle indicated the results obtained by hub miRNAs. (B) Comparisons of miRNA signature prognostic power in GBMs.

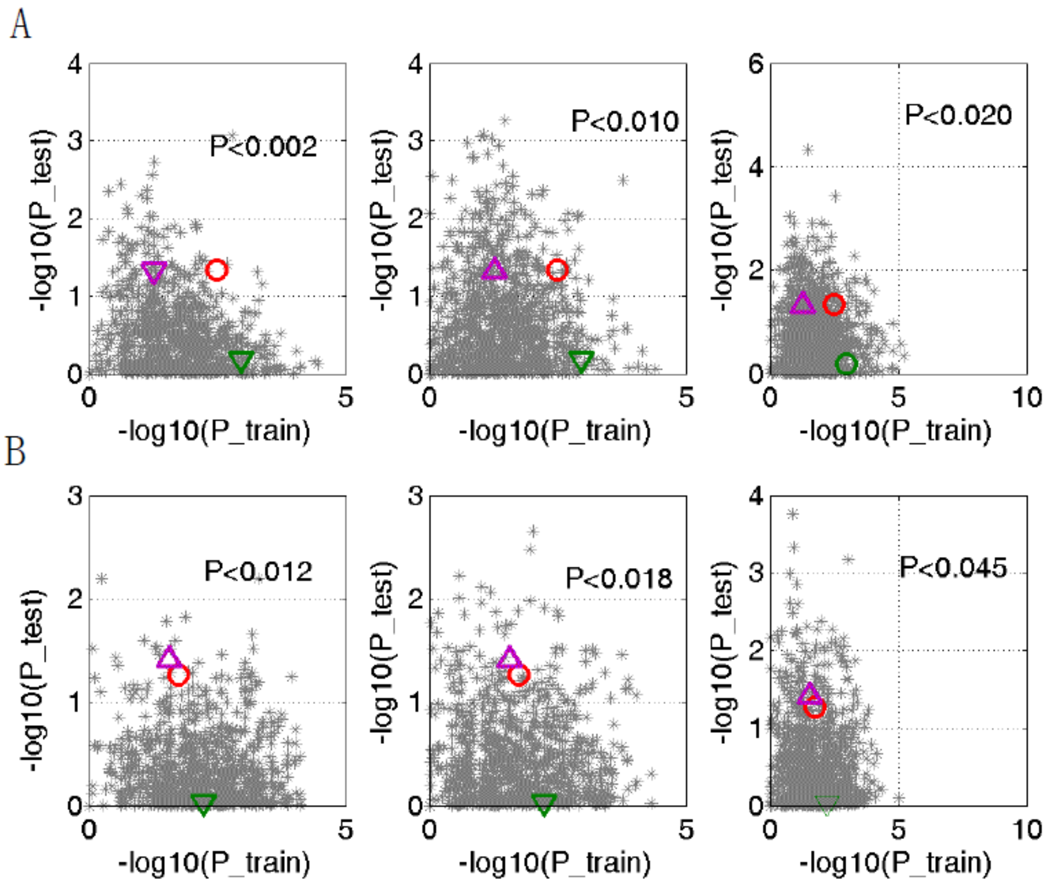

Supplementary Figure S5. Network-based approach increases the power to identify the miRNA prognostic signature (top 20% as hubs).

(A) Comparisons of miRNA signature prognostic power in high-grade gliomas. Left panel, random miRNAs from non-hub miRNAs in the FMRN; middle panel, random miRNAs from differentially expressed miRNAs; right panel, random miRNAs from all miRNAs in the microarray. The green triangle is based on the most differentially expressed 30 miRNAs in type III, while the pink triangle is based on the most differentially expressed 30 miRNAs in GBMs. The red circle indicated the results obtained by hub miRNAs. (B) Comparisons of miRNA signature prognostic power in GBMs.

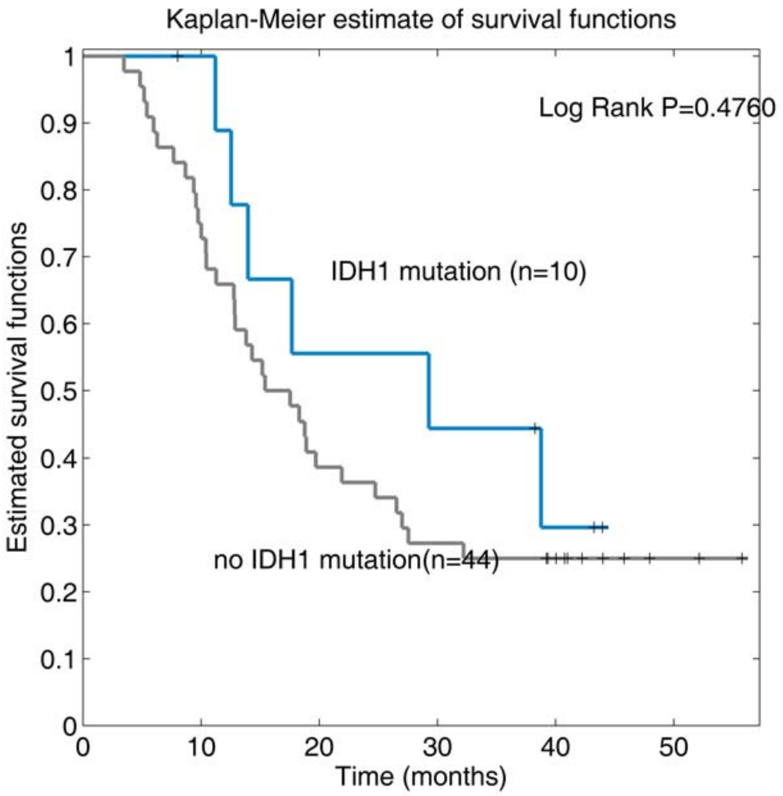

Supplementary Figure S6. Kaplan-Meier curve of overall survival according to the IDH1 mutation status in the GBM patients.

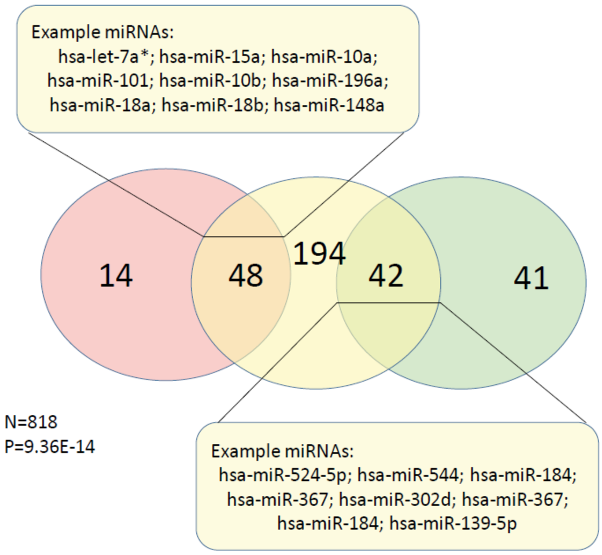

Supplementary Figure S7. Most of the miRNAs in the FMRN were reported to be associated with glioma in previous studies.

Here the known miRNAs were collected from the review of Duroux et al.
